# Chemical and structural characterization of hemicellulose from date fruits (*Phoenix dactylifera* L.)

**DOI:** 10.3389/fnut.2026.1804879

**Published:** 2026-03-26

**Authors:** Clinton E. Okonkwo, Matthew J. O’Connor, Yue Ling Wong, Laura Nyström, Ali H. Al-Marzouqi, Mutamed Ayyash, Dorin Boldor, Afaf Kamal-Eldin

**Affiliations:** 1 Department of Food Science and Technology, College of Agriculture and Veterinary Medicine, United Arab Emirates University, Al Ain, United Arab Emirates; 2 Core Technology Platforms, New York University Abu Dhabi, Abu Dhabi, United Arab Emirates; 3 ETH Zürich, Institute of Food, Nutrition and Health, Schmelzbergstrasse 9, Zurich, Switzerland; 4 Department of Chemical and Petroleum Engineering, United Arab Emirates University, Al Ain, United Arab Emirates; 5 Department of Biological and Agricultural Engineering, Louisiana State University Agricultural Center, Baton Rouge, LA, United States

**Keywords:** arabinogalactan-protein, arabinoxylan, date fruits, hemicellulose, rhamnogalacturonan-I

## Abstract

In this study, we extracted hemicellulose from two date fruit cultivars (soft Barhi and hard Neghal) and investigated their structural, physicochemical, and thermal properties. Alkali solubilized more hemicellulose (45%–47.5%) than DMSO (6%–7%); however, DMSO preserved the feruloyl ester linkages. The monosaccharides constituent of hemicellulose includes xylose (33.1–70.6%), glucose (6.7%–17.2%) uronic acids (5.5%–13.3%), arabinose (3.2%–10.1%), galactose content (4.8%–6.8%), mannose (1.6%–3.6%), and rhamnose (0%–3.1%). The hemicellulose was primarily *β*–(1 → 4)–linked arabinoxylans with minor glucurono−/galacto-arabinoxylan motifs, together with insoluble glucan and pectic homogalacturonan (HG) and rhamnogalacturonan-I (RG-I). Neghal hemicellulose was structurally simpler, containing relatively less branched arabinoxylan and showing lower peak-molecular weights (27.5–35 kDa), whereas Barhi hemicellulose was enriched in arabinogalactan-protein (AGP) and RG-like features and showed higher peak-molecular weights (29.5–223.9 kDa). These structural differences correlate with a more branched, hydrated and less tightly packed hemicellulose–pectin matrix in Barhi compared with a more dominated arabinoxylan–matrix in Neghal and may contribute to the softer texture of Barhi. Overall, these results provide new insights into the composition and structural diversity of alkali-soluble hemicellulosic polysaccharides in date fruits.

## 1 Introduction

Hemicellulose represent a large group of complex polysaccharides that, after cellulose, represent the second major component of plant cell walls (1). Unlike cellulose, they comprise branched polymers (20–200 kDa) containing various monomeric pentoses (xylose and arabinose), hexoses (mannose, glucose, galactose), and sometimes uronic acids (2). Structurally, hemicellulose can be classified into xylan, xyloglucan, mannan, and mixed-linkage β-glucan (3, 4). Generally, hemicellulose is attached to cellulose microfibrils *via* hydrogen bonds and cross-link between cellulose microfibrils (1). On the other hand, hemicellulose is partly linked to lignin *via* ferulate or di-ferulate residues, which are ester-linked to hemicellulose and ether-linked to lignin (5, 6). This structural complexity makes it challenging to investigate the utilization and properties of hemicellulose. Moreover, there has been growing trend in the utilization of hemicellulose in the food industry, as well as in packaging, cosmetics, and pharmaceuticals (2).

Date fruits (*Phoenix dactylifera* L.) belong to the Arecaceae family (7) and are native to the Middle East and North Africa (8). Date fruits are consumed fresh, dried or processed into syrup, paste, jam, vinegar, sugar, etc. (9, 10), and offers several health benefits including antioxidant, anticancer, antidiabetic, anti-inflammation, and immune-modulation (11, 12). For this reason, there has been great interest in its chemical composition, especially its dietary fiber. Date fruit dietary fiber is primarily insoluble fiber (85%–92%), comprising cellulose (24%–38%), hemicellulose (28%–43%), and lignin (23%–40%) (13–15). Till date, little is known about the date fruit hemicellulose, even though there have been studies on hemicellulosic polysaccharides from other palm fruits of the Arecaceae family. For instance, galactomannoglucan was identified in *Arecastrum romanzoffianum* (16); acetylated 4-O-methylglucuronoarabinoxylan in *Elaeis guinensis* (17); linear (1 → 4)-*β*-ᴅ-xylan with linear (1 → 5)-*α*-L-arabinan and (1 → 3)-(1 → 4)-α-ᴅ-glucan in *Mauritia flexuosa* (18); linear (1 → 4)-β-ᴅ-xylan in *Euterpe oleraceae* (19); and acidic galactoarabinoxylan with a linear (1 → 5)-α-L-arabinan, a low branched glucuronoxylan, and small amount of xyloglucan in *Astrocaryum aculeatum* (20).

To study and utilize the hemicellulose in date fruits, extraction is a necessary step. Several methods can be used to extract hemicellulose from plants, including chemical (alkaline, alkaline peroxide, organic solvent) and biological (enzymes) approaches (21). Among these, alkaline extraction remains prevalent due to its high yield, low cost, preservation of a high degree of polymerization, and low hydrophilicity (22–24), although it may cause deacetylation of the hemicellulose (21). Therefore, this study focused on extraction of hemicellulose from two date fruit cultivars with varying hardness (soft Barhi and hard Neghal), including the determination of molecular weight, elucidation of the chemical structures, and evaluation of their microstructure and thermal properties, which will facilitate its onward utilization.

## 2 Materials and methods

### 2.1 Chemicals and reagents

Analytical grade sodium hydroxide, dimethyl sulfoxide (DMSO), ethanol, acetone, sodium chlorite, glacial acetic acid, sulfuric acid, ammonium hydroxide, potassium borohydride, 1-methyl imidazole, fucose, galactose, glucose, mannose, arabinose, rhamnose, xylose, acetonitrile, methanol, formic acid, sodium nitrate, sodium azide, maltotriose, and maltohexaose were purchased from Sigma Aldrich (St Louis, MO, USA). Maltoctaose was purchased from Megazyme (Bray, Ireland). Pullulan standards were purchased from Shodex (Munich, Germany).

### 2.2 Samples and texture analysis

Two date cultivars, soft (Barhi) and hard (Neghal), were selected as representative soft and hard varieties because they exhibit contrasting texture and dietary fiber content and composition as shown in previous studies (14, 25). The Barhi and Neghal cultivars were collected from Al-Foah Date Factory (Al Ain, United Arab Emirates). A computerized CT3 texture analyzer equipped with a 4.5 kg load cell (TA instruments, Middleboro, MA, USA) was used to analyze the texture of the date fruits (Barhi and Neghal cultivars). All experiments were conducted at 25 °C ± 2 °C with a 2 mm diameter penetration probe. One pitted date was divided into two equal halves, one side was placed over the other, and the middle of the date halves were penetrated at a 5 mm target value at a compression rate of 1 mm/s. Stacked halves was used to reduce curvature and obtain a flat, well-defined contact surface, and to ensure that the probe compressed tissue of comparable thickness across cultivars. Furthermore, the cut surfaces were cautiously aligned, a pre-load was applied to ensure full contact, and samples exhibiting visible gaps or slippage were not used. The middle of the date halves was selected as the measurement location to avoid edge regions and based on preliminary trials, which showed lower variability and fewer edge effects at this location. All measurements were performed with 15 replications per cultivar., each measured using the same stacked-halves protocol (25).

### 2.3 Sample preparation and insoluble fiber extraction

Desugaring and insoluble fiber extraction from date fruits were performed as described in our earlier study (15). Desugared fibers were treated with an ethanol: acetone solution (1:2 v/v) at 85 °C for 8 h to remove extractives (4). The extractive-free residues were mixed with distilled water (1:14 w/v) and heated in a water bath (WSB-18, Witeg Labortechnik GmbH, D-97877, Wertheim, Germany) at 95 °C for 15 min and then at 60 °C for 1 h with constant shaking at 200 rpm. The resultant mixture was vacuum filtered, washed three times with hot water (70 °C), and the procedure was repeated to more effectively remove the soluble fiber fraction. The insoluble residues were washed with ethanol (78% v/v and 95% v/v), treated with acetone twice, and oven-dried (FD-S 056, Binder GmbH, Im Mittleren Ösch 5, 78532 Tuttlingen, Germany) at 50 °C for 18 h (15, 26).

### 2.4 Hemicellulose extraction

**Figure 1:**
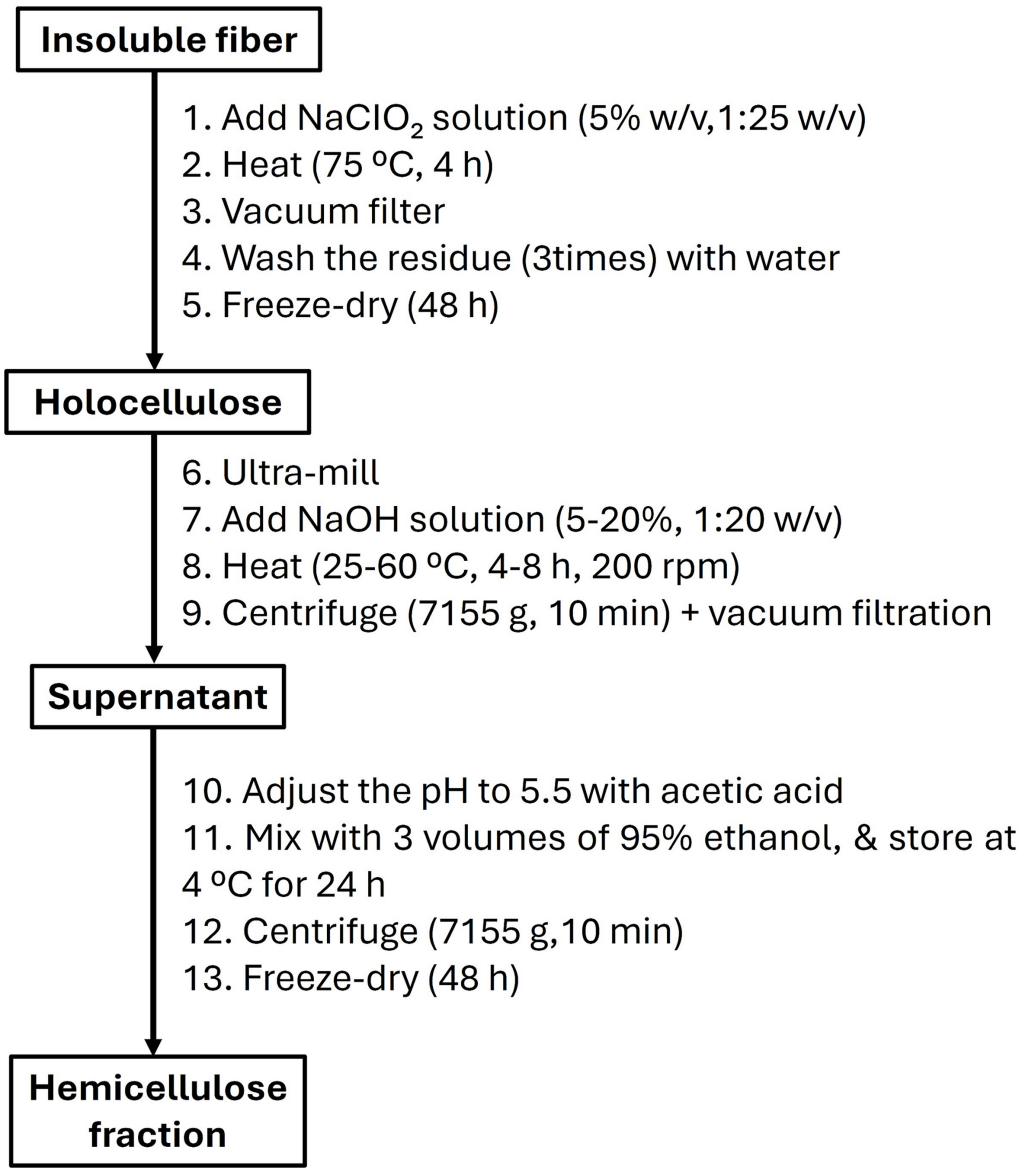
Hemicellulose extraction.

The insoluble fibers were mixed with sodium chlorite solution (5% w/v, solid-to-liquid ratio 1:25 w/v) and heated (75 °C) for 4 h. The reaction mixtures were vacuum filtered, and the solid residues were washed three times with deionized water and freeze-dried (Bioevopeak, LYO60B-1P, Shandong, China) to obtain holocellulose (Figure 1). The holocellulose was ground with an ultra-centrifugal mill (ZM 200, Retsch GmbH, Retsch-Allee 1–542781 Haan, Germany), dissolved in NaOH solution (5%–20% w/v, solid-to-liquid ratio 1:20 w/v), and heated in a water bath (25 °C–60 °C, 4–8 h, 200 rpm). The mixtures were then centrifuged (Digicen 21R, Orto alresa, Misericordia, 2328864, Ajaivir, Madrid, Spain) at 7155 g for 10 min and vacuum-filtered to separate the hemicellulose-rich fraction from the solid residue (4). The solid residue was washed twice with deionized water to remove any residual hemicellulose, and the filtrates were combined. The pH of the hemicellulose-rich filtrates was adjusted to 5.5 using acetic acid, and ethanol (95% v/v), equivalent to three times the volume of the filtrates, was added to the mixtures and allowed to stand in a refrigerator (4 °C) for 24 h to precipitate hemicellulose. Finally, the mixtures were centrifuged (7,155 g) for 10 min and freeze-dried for 48 h to obtain the dried hemicellulose fraction, and the yield was estimated with Equation 1. Dried hemicellulose from the Barhi and Neghal cultivars were stored in a desiccator for further characterization. Also, hemicellulose was extracted from Barhi and Neghal holocellulose with DMSO (80% v/v) in a water bath (200 rpm, 80 °C, 8 h, 1:20 w/v sample-to-solvent ratio), as a comparative extraction method. DMSO solubilizes hemicelluloses under relatively mild conditions with limited additional degradation, whereas NaOH efficiently extract hemicelluloses by breaking lignin–carbohydrate bonds, even though it is more chemically aggressive (24).


$$\text{Yield}=\frac{\text{weight of dried hemicellulose}\;}{\text{initial weight of holocellulose}}\times 100$$ (1)

### 2.5 Response surface methodology

The suitable ranges of independent variables (alkali concentration, heating time, and temperature) required for hemicellulose and cellulose separation were selected based on preliminary single factor experiments and previous reports (1, 4). In the preliminary trials we varied each factor over a wider interval and monitored hemicellulose yield and monosaccharides composition. Subsequently, response surface methodology was used to analyze the combined effect of the three variables on hemicellulose extraction yield. A Box–Behnken design was used to determine the optimal conditions for hemicellulose extraction. The ranges of independent variables were selected and coded using Equation 2.


$$X_{i}=\frac{x-(x_{a}+x_{b})/2}{(x_{a}-x_{b})/2}$$ (2)

Where X_i_, x, x_a_, and x_b_ represent coded value of the independent variable i, actual value of the independent variable, actual value at the center point of the range (mean), and step change (half of the total range) used to convert from actual to coded units, respectively. The minimum and maximum levels of alkaline concentration (5%–20%), heating time (4–8 h), and temperature (25 °C–60 °C) were established. Three factors, three levels, and three center points in Box–Behnken design generated a total of 15 runs (Table 1).

**Table 1:** Hemicellulose extraction conditions.

S/N	Alkali concentration (%)	Heating temperature (°C)	Heating time (h)
1	20.0	60.0	6
2	12.5	25.0	4
3	5.0	42.5	4
4	20.0	42.5	8
5	5.0	25.0	6
6	12.5	60.0	8
7	12.5	42.5	6
8	12.5	42.5	6
9	5.0	42.5	8
10	12.5	42.5	6
11	12.5	25.0	8
12	5.0	60.0	6
13	12.5	60.0	4
14	20.0	25.0	6
15	20.0	42.5	4

Coded variables (−1, 0, +1).

The data obtained from this experiment were evaluated through multiple regression to fit the second-order polynomial (Equation 3).


$$Y=\beta_{O}+\sum_{i=1}^{n}\beta_{i}X_{i}+\sum_{i<j}^{n}\;\beta_{\mathrm{ij}}X_{i}X_{j}+\sum_{i=1}^{n}\beta_{\mathrm{ii}}X_{i}^{2}$$ (3)

Where Y represents the response or dependent variable (yield of hemicellulose), *n* is the number of variables, β_o_, β_i_, β_ij,_ and β_ii_ are constant coefficients for intercept, linear, interaction, and quadratic effects, respectively. X_i_ and X_j_ represent the levels of independent variables. Model selection was based on the F-test, lack-of-fit test, and coefficient of determination (R^2^).

### 2.6 Ferulic acid analysis

The hemicellulose fractions from alkaline and DMSO extractions were semi-quantitatively analyzed for the presence of ferulic acid according to methods used by Pellati et al. (27) and Pazo-Cepeda et al. (28) with slight modifications. Briefly, 250 mg of sample was mixed with NaOH (3 M, 15 mL), sealed with aluminum foil, and allowed to stand for 3 h in a dry-dark cupboard. The mixture was centrifuged (7,155 g) for 10 min, and the pH of the supernatant was adjusted to 2 with a few drops of concentrated HCl. The supernatant was then extracted three times with ethyl acetate, and the organic phase was collected, vacuum evaporated (Rotavapor, R-300, BUCHI Labortechnik AG, Switzerland) to dryness, reconstituted in methanol (3 mL), filtered (0.45 μm), and analyzed in a Shimadzu chromatography system (Shimadzu HPLC, Kyoto, Japan). Shimadzu HPLC included a vacuum degasser, a pump, an autosampler, a column compartment, and an SPD-M20A diode array detector (DAD). The analysis was performed using an Ascentis C_18_ column (250 mm × 4.6 mm I.D., 5 μm, Supelco, Bellefonte, PA, USA). The mobile phase consisted of water (A) and acetonitrile (B) with 0.1% formic acid. The gradient elution was as follows: 0–3 min, 25% B; 3–10 min, 25 to 27% B; and 10–30 min, from 27% to 40% B. The post-run time was 5 min, with a flow rate of 1.0 mL/min, column temperature of 30 °C, a sample injection volume of 5 μL, and DAD acquisition at 320 nm. Ferulic acid standard was prepared and ran alongside the samples.

### 2.7 Monosaccharides composition

Monosaccharide composition of hemicellulose fractions was analyzed using the Uppsala method (29). The sample was hydrolyzed using a two-step acid hydrolysis method: H_2_SO_4_ (12 M, 3 mL) was added to the sample (0.1 g) and incubated in a water bath (WB-11, Witeg Labortechnik GmbH, D-97877, Wertheim, Germany) at 30 °C for 1 h, diluted with deionized water (74 mL), and myo-inositol standard (2 mg/mL, 10 mL) was added. The mixture was then autoclaved (CL-40 L, ALP Co., Ltd., Midorigaoka, Hamura-shi, Tokyo, Japan) at 125 °C for 1 h. Alditol acetates were extracted from different sugars using ethyl acetate. The mixture was vacuum-filtered, and the hydrolysate was derivatized (reduction and acetylation) and analyzed in the gas chromatography instrument (YL Instrument, 6500GC System, Hogye-dong, Anyang, Korea). A mixture of sugar standards (glucose, rhamnose, arabinose, xylose, fucose, galactose, and mannose) and an internal standard (myo-inositol) were prepared and subjected to the same hydrolysis and derivatization steps. Samples and standards were injected using an autosampler in split mode with an injection volume of 1.0 μL and a split ratio of 10:1. The gas chromatography system was integrated with gas generators (oxygen, nitrogen, and hydrogen), a capillary column (15 m × 0.25 mm id, 0.15 μm film thickness, DB 225), a split mode injector (ratio, 1:50), and a flame ionization detector. The temperature program was set to 160 °C (6 min initial), increased to 220 °C at 4 °C/min and held at 220 °C in 4 min. Nitrogen was used as carrier gas (10 mL/s), and zero air (300 mL/s) and hydrogen (30 mL/s) were used as the combustion gases. An internal standard, myo-inositol (2 mg/mL) was added to calibration standards and sample extracts prior to derivatization, and samples or calibration standards with recoveries not in the range of 80%–100% were re-analyzed. Each extract was injected three times, checked for outliers, and mean and standard deviation was reported. A multi-point calibration curve (7 concentration levels) was used for each analyte by plotting the analyte-to-internal-standard peak area ratio against concentration, and linearity was analyzed with coefficient of determination (*R*^2^ ≥ 0.99). Also, solvent blank (ethyl acetate) was injected at the start and in between batches to check for carryover and contamination.

Uronic acid in the hydrolysate was also analyzed by mixing hydrolysate (250 μL) with boric acid-sodium chloride solution (250 μL) and sulfuric acid (18 M, 4 mL), vortexing, and incubating in a water bath (70 °C, 40 min) (29). Following cooling, dimethylphenol solution (200 μL) was added, vortexed, and the absorbance was measured against a blank (10–25 min after adding dimethylphenol) at wavelengths of 400 nm and 450 nm using a UV–visible spectrophotometer (Shimadzu, Columbia, Maryland, USA). The absorbance at 400 nm was subtracted from absorbance at 450 nm to correct for interference of hexoses. The corrected absorbance values were then used to prepare the calibration curve, and the uronic acid concentration in the samples was calculated from this curve (29).

Protein content in the hemicellulose samples was determined by Kjeldahl method. Samples were digested overnight (Büchi Digest System K-437, Switzerland), after which nitrogen content was determined in an AutoKjeldahl unit (Buchi AutoKjeldahl Unit K-370, Flawil, Switzerland) and then converted to total protein using a nitrogen conversion factor of 6.25. Analysis was conducted in triplicate and values expressed as mean ± standard deviation.

### 2.8 Nuclear magnetic resonance spectroscopy

Nuclear magnetic resonance (NMR) spectroscopy analysis was conducted on two (2) hemicellulose samples (5%/6 h/60 °C and 12.5%/8 h/25 °C) from Barhi and Neghal in D_2_O at 60 °C using a 600 MHz NMR with cryoprobe. The ^1^H NMR spectrum was recorded using zgpr to suppress the residual water signal with 128 scans, four dummy scans, and a d1 set to 5 s. The ^13^C NMR spectrum was taken with standard proton decoupled NMR for 8,192 scans. Proton correlation spectroscopy (^1^H COSY) and proton total correlation spectroscopy (^1^H TOCSY) NMR were used to determine structural information on carbohydrate rings. COSY used the cosygppppf F1 program with 128 points and 32 scans, while TOCSY used the mlevphpp F1 program with 256 points and 64 scans. Heteronuclear single quantum correlation (HSQC) NMR was used to enhance resolution and find basic, rudimentary assignments. The HSQC program hsqcedetgpsisp2.3 from the Bruker spectrum library (F1) used 256 points and 64 scans. Heteronuclear multiple-bond correlation (HMBC) NMR determined long-range couplings indicative of linkage types and branches in polysaccharides, using the hmbcetgpl3nd program from the Bruker library (F1) with 256 points, 64 scans, and a dwell time of 50 μs. The plots were processed using MestReNova software.

### 2.9 Molecular weight determination by high-performance size-exclusion chromatography (HPSEC)

The molecular weight of hemicellulose was determined using HPSEC (30). Samples (20 mg) were dissolved in 0.1 M NaNO_3_ containing 0.02% w/v NaN_3_ (10 mL) to achieve a final concentration of 2 mg/mL and filtered (PTFE, 0.45 μm). The HPSEC system included a binary pump, degasser, thermostated column compartment, auto sampler, DAD, and refractive index detector, all sourced from Agilent Technologies (Series 1,200, California, USA). The column system consisted of a pre-column (Suprema 50 mm × 8 mm, 10 μm, PSS Polymer Standards Service GmbH, Germany) connected to a Suprema 30,000 column (10 μm, 8 mm × 300 mm, PSS Polymer Standards Service GmbH, Germany) and an A5000 column (Viscotek, 300 mm × 7.8 mm, Malvern Instruments Ltd., UK) in series as recommended by the manufacturer. Malvern Instruments Ltd., UK) in series. The temperature of the column compartment was kept at 35 °C, the flow rate was 1 mL/min and the injection volume 50 μL. For the calibration curve, 2 mg/mL solutions of pullulan standards with peak-molecular weights of 5,900, 9,600, 21,100, 47,100, 107,000, 200,000, 344,000, and 708,000 g/mol (Shodex, Munich, Germany), as well as maltotriose, maltohexaose, and maltooctaose were prepared in the same eluent and filtered (PTFE, 0.45 μm). Molecular weight calibration was carried out by plotting elution volume against log peak–molecular weights of the standards, and values are expressed as pullulan–equivalent molecular weights (M_p_). Moreover, due to the differences in hydrodynamic properties between pullulan and hemicellulose, these values should be regarded as relative rather than absolute molecular weights. Also, the column set was validated by calibration with pullulan standards over the full molecular weight range, yielding a linear calibration (*R*^2^ ≥ 0.99) and no indication of peak distortion or loss of resolution at the overlap between columns.

### 2.10 Light and scanning electron microscopy

A professional trinocular stereo zoom microscope (AmScope SM-3T, Ningbo, Zhejiang province, China) was used to capture images of hemicellulose fractions at 20 × magnification. The morphology of hemicellulose fractions was examined using scanning electron microscopy (JEOL Ltd., Tokyo, Japan). Samples were mounted on a specimen stub using carbon adhesive tape and coated with *gold* (80 s at 40 mA). Images were captured at different magnifications (1,000×, 500×, and 150×).

### 2.11 X-ray diffraction

An X-ray diffractometer (X’Pert PRO MRD XL XRD System from PANalytical, EA Almelo, Netherlands) was used to analyze the hemicellulose fractions using Cu K-α radiation at a wavelength of 1.54Å, with a minimum step size of 2θ.

### 2.12 Thermal properties

The thermal properties of hemicellulose fractions were analyzed using a thermogravimetric (TG) analyzer (TGA Q500, TA Instruments, Delaware, USA). The heating rate was set at 20 °C/min, with a nitrogen flow rate of 50 mL/min. Samples (10 mg) were heated from 20 °C to 700 °C (15). The thermogravimetric and derivative thermogravimetry (DTG) curves were plotted.

### 2.13 Statistical analysis

Response surface modeling of the hemicellulose yield was performed using Design-Expert Version 13.0.5.0 (Stat-Ease Inc., Minneapolis, MN, USA), and model suitability was determined from the F-test, *p*-value (*p* < 0.05), lack-of-fit test, and coefficient of determination (*R*^2^). Principal component analysis (PCA) was used to analyze the multivariate relationships in the monosaccharides data among cultivars and extraction conditions using OriginPro 2025b (OriginLab Corporation, Northampton, USA). PCA was conducted on the data after mean-centering and autoscaling of variables to unit variance. The number of components retained for interpretation was based on examining the scree plot, cumulative explained variance, and eigenvalues > 1. Model robustness was analyzed using leave-one-out / k-fold cross-validation, and only components that were steady under cross-validation were studied. Scores and loading plots were investigated together to elucidate group separation and variable contributions. An independent t-test was used to compare the mean texture values (*n* = 15) in OriginPro 2025b (OriginLab Corporation, Northampton, MA, USA), and data was presented as mean ± standard deviation at a significance level of *p* < 0.05. Hemicellulose yields of both cultivars from alkaline (at optimum) and DMSO extraction were analyzed using two-way ANOVA, followed by Tukey’s *post hoc* test for pairwise comparisons of means at a significance level of *p* < 0.05. Monosaccharide analyses were conducted in triplicate and data expressed as mean ± standard deviation, and two-way ANOVA was used to assess the effects and interactions, followed by Tukey’s *post hoc* test for pairwise comparisons of means at a significance level of *p* < 0.05. Graphs were plotted using OriginPro 2025b (OriginLab Corporation, Northampton, MA, USA).

## 3 Results and discussion

### 3.1 Date fruit texture

Compared with Neghal, the Barhi dates showed significantly (*p* < 0.05) lower hardness (189 g vs. 483 g), gumminess (93.5 g vs. 241 g) and chewiness (3.3 mj vs. 7.9 mj), but slightly higher adhesiveness and springiness, and similar cohesiveness (Table 2). These results confirm that Barhi dates represent the softer cultivar and Neghal the harder cultivar., providing a distinct textural difference for succeeding comparison of hemicellulose characteristics. This texture profile approximates the whole-fruit behavior; however, the curvature and seed of intact fruit may contribute additional mechanical responses that are not captured by our protocol.

**Table 2:** Texture analysis of date fruits (Barhi and Neghal cultivars).

Parameters	Barhi	Neghal
Hardness (g)	189 ± 28.5^a^	483 ± 56.9^b^
Adhesiveness (mj)	0.9 ± 0.3^a^	0.2 ± 0.2^a^
Cohesiveness	0.5 ± 0.1^a^	0.5 ± 0.1^a^
Springiness (mm)	3.8 ± 0.8^a^	3.3 ± 0.2^a^
Gumminess (g)	93.5 ± 24.5^a^	241.0 ± 49.3^b^
Chewiness (mj)	3.3 ± 0.7^a^	7.9 ± 1.9^b^

*n* = 15. Different letters in different columns indicate significance differences (*p* < 0.05) between Barhi and Neghal cultivars, while the same letters represent non-significant differences (*p* > 0.05).

### 3.2 Hemicellulose yield

**Figure 2:**
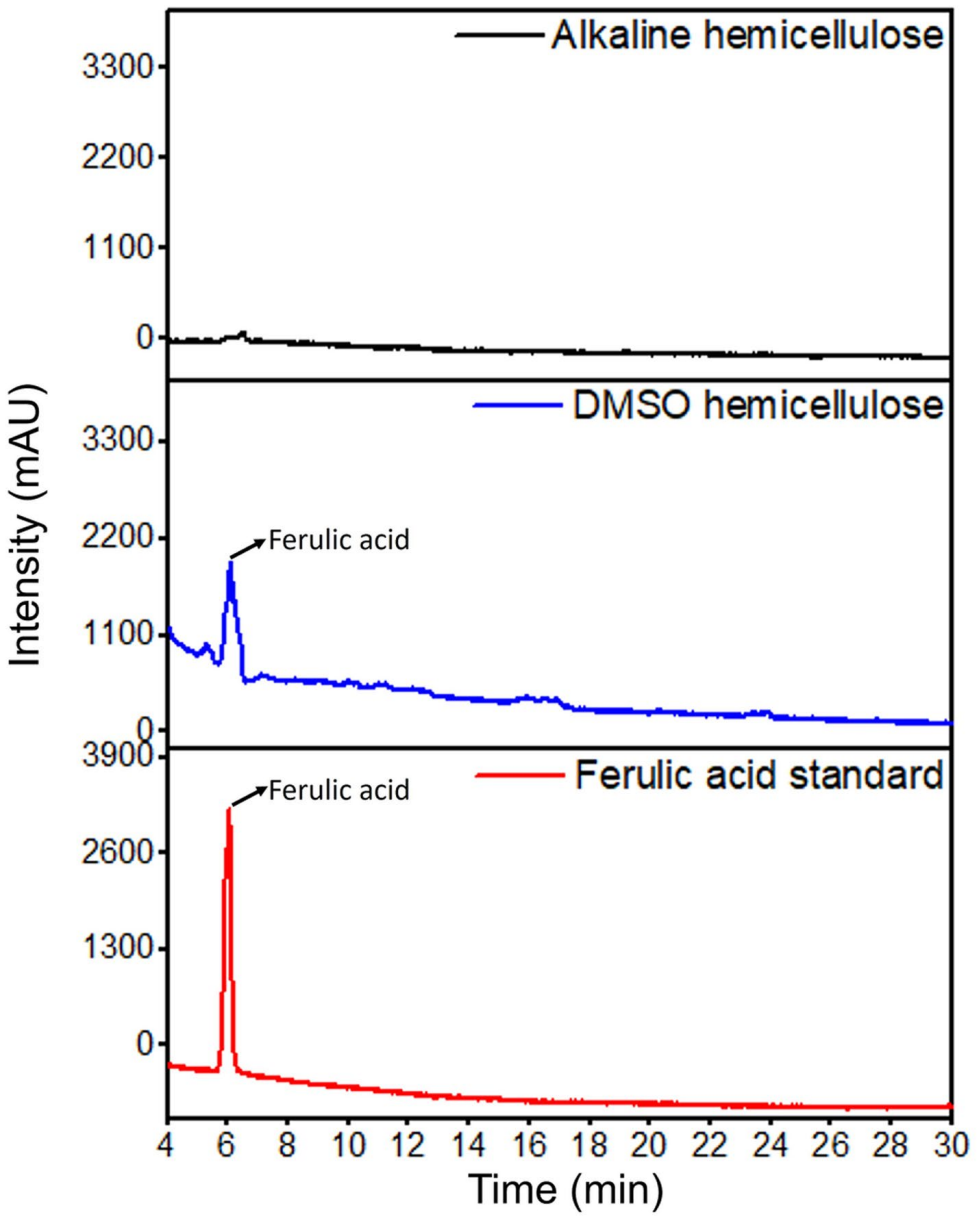
Presence of ferulic acid in the hemicellulose.

Alkali (20%, 8 h, 60 °C) extracted significantly (*p* < 0.05) higher hemicellulose yield (45%–47.5%) than DMSO (6%–7%) (Supplementary Figure S1). This difference is due to the ability of NaOH to cleave ester linkages that bind hemicellulose to lignin *via* ferulate (21, 31). The carboxyl group of ferulic acid is esterified at the O-5 position of arabinose residues in arabinoxylan (1, 32, 33). The absence of ferulic acid in alkaline-extracted hemicellulose (Figure 2) aligns with previous studies (1, 34), suggesting that the breakage of these ester bonds facilitates the extraction of this type of hemicellulose once it is liberated from its association with lignin (1, 31). Fruit skin tissues have been shown to contain ferulate esterified to polysaccharides (35). In date fruit, guaiacyl lignin units are deposited in the sclerenchyma and parenchyma cell walls while syringyl lignin units are found in the secondary walls of the xylem vessels (15).

**Figure 3:**
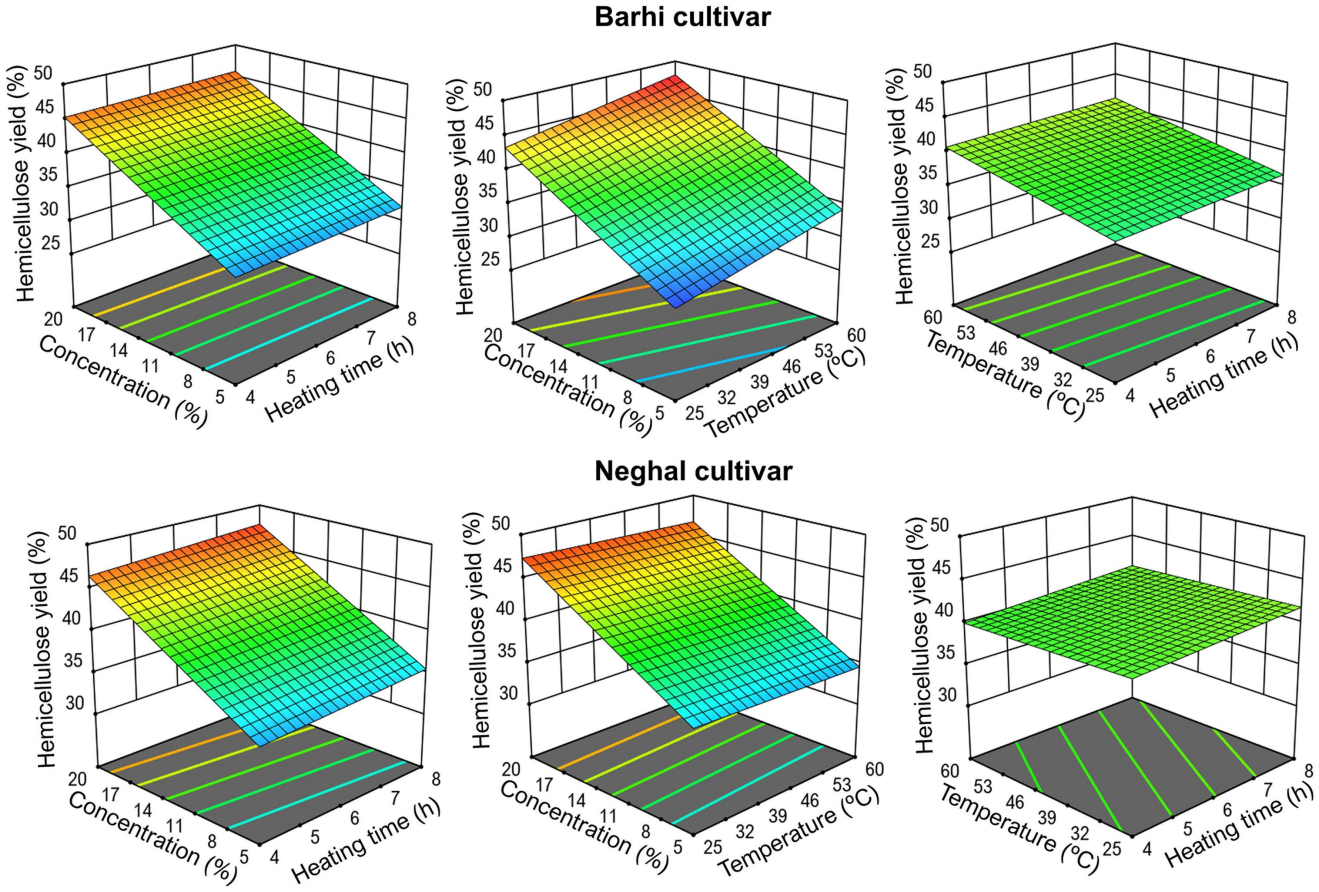
Hemicellulose yields from alkaline extractions.

Increasing alkali concentration (from 5% to 20% w/v) significantly enhanced hemicellulose yield from 25% to 47.5% (*p* < 0.05, Supplementary Table S1), whereas increasing extraction temperature and heating time had minimal impact on hemicellulose yield (Figure 3; Equations 4, 5). Strong alkali produces more hydroxyl ions, causing cellulose to swell and disrupting the strong hydrogen bonds between hemicellulose and cellulose, thereby enhancing hemicellulose extraction (31, 36). Ghosh et al. (37) stated that the increasing heating temperature and time can enhance diffusion and reaction rates but may not significantly increase yield once the system is saturated with alkali. Yang et al. (38) noted that compared to alkali concentration, reaction time had a lesser effect during hemicellulose extraction. For Barhi holocellulose, temperature rise slightly increased hemicellulose yield, whereas for Neghal holocellulose, temperature rise slightly reduced the hemicellulose yield (Equations 4, 5). These differences might be due to the inherent characteristics of the plant cell wall. In plants, hemicellulose mainly exists in the secondary cell wall and parenchyma cells; however, those in parenchyma cells are more easily solubilized than those in the secondary cell wall (23, 39). Dense vascular bundle structures and low parenchyma cell are associated with reductions in the hemicellulose extraction rate (23).

Linear models were selected to explore the relationship between hemicellulose yield, alkali concentration, heating time, and temperature due to their higher R^2^ and lower *p*-value (Supplementary Table S2).


Barhi hemicellulose yield=20.93+0.91A+0.13B+0.13C (4)

Neghal hemicellulose yield=29.81+0.81A+0.30B−0.02C (5)

Where A, B, and C represent alkali concentration (5%–20%), heating time (4–8 h), and temperature (25 °C–60 °C). Thus, the alkali concentration is more effective in determining the yield than the extraction time and temperature. The harshest extraction conditions are expected not only to affect the yield but also the composition and properties of the extracted hemicellulose (See below). It is difficult to correlate extracted hemicellulose with texture because mild conditions might extract a non-representative proportion while harsh conditions might modify its structure.

### 3.3 Monosaccharides composition of the hemicellulose

Compared to the Barhi cultivar., the Neghal cultivar hemicellulosic fraction (*p* < 0.05) contained more xylose (52.8%–70.6% vs. 33.1%–45.3%) and uronic acids (8.3%–13.3% vs. 5.5%–9.1%), but lower glucose (7.4%–15.6% vs. 6.7%–17.2%), arabinose (3.2%–4.8% vs. 5.8%–10.1%), mannose (1.6%–3.2% vs. 2.8%–3.6%), and rhamnose (0 vs. 0%–3.1%), with similar galactose content (5.3%–6.8%) (Table 3; Supplementary Table S3). Similar monosaccharides were found in hemicellulose from other fruits: *Mauritia flexuosa* (18), *Euterpe oleraceae* (19), *Astrocaryum aculeatum* (20), and *Campomanesia pubescens* (40). These findings suggest that date fruit hemicellulose is mainly xylan-type polysaccharides with lower amounts of glucan, arabinan, galactan, arabinogalactan, and pectic substances. The higher xylose content in Neghal hemicellulose compared to Barhi indicates that Neghal’s hemicellulosic fraction possesses more xylan-type polysaccharides, and these xylans, through their arabinose side chains, associate with lignin through ferulic acid bridges, contributing to the hardness of Neghal fruit compared to Barhi. Weak alkali extracted more xylose, uronic acid, and rhamnose, whereas strong alkali extracted more glucose and mannose (Table 3). Nishitsuji et al. (31) reported that, for wheat, strong alkali solubilizes more glucose, whereas weak alkali solubilizes more galactose and mannose. These differences may relate to variations in plant cell wall composition or the alkaline concentration range. The uronic acid/xylose (UA/Xyl) and arabinose/xylose (Ara/Xyl) ratios can be used to evaluate the degree of branching or linearity of hemicellulose (41). A high Ara/Xyl ratio indicates a more branched/substituted hemicellulose, while a low Ara/Xyl ratio suggests lesser branching (1). Barhi cultivar showed greater Ara/Xyl ratio (0.16–0.27) compared to Neghal hemicellulose (0.05–0.08), indicating higher degree of branching/substitution in arabinoxylan-type hemicellulose (Table 3; Supplementary Table S3). Increased substitution typically enhances hydrophilicity and water-binding capacity, which may reduce tight packing with cellulose and other polymers in the cell wall, thereby promoting a more hydrated and plastically deformable cell wall matrix (42, 43). In our data, Barhi cultivar with higher Ara/Xyl ratio also showed lower hardness and chewiness (Table 2), which is consistent with the mechanistic explanation. The high alkali concentration, elevated temperature, and longer heating durations extracted hemicellulose with a higher degree of branching. This may be due to strong alkali cleaving more ester bonds, facilitating the dissolution of arabinoxylan with a higher Ara/Xyl ratio (31, 34). The UA/Xyl ratio in Barhi hemicellulose (0.13–0.26) was similar with UA/Xyl ratio in Neghal hemicellulose (0.14–0.20) (Table 3; Supplementary Table S3), indicating a comparable degree of uronic acid substitution on the xylan backbone.

**Table 3:** Monosaccharides composition of alkali-extracted hemicelluloses (Subset of Supplementary Table S3).

Alkali conc. (%), time (h), temp. (°C)	Rhamnose (%)	Mannose (%)	Galactose (%)	Arabinose (%)	Xylose (%)	Glucose (%)	Uronic acid (%)	Arabinose/Xylose ratio	Uronic acid/Xylose ratio
Barhi									
12.5, 8, 25	n.d.	3.2 ± 0.75^cb^	5.1 ± 0.33^ac^	6.5 ± 0.37^b^	36.9 ± 0.68^e^	14.1 ± 0.97^kb^	6.0 ± 0.34^cd^	0.17	0.16
5, 6, 60	3.1 ± 0.16^c^	n.d.	6.5 ± 0.29^s^	10.1 ± 1.52^d^	43.6 ± 1.36^n^	6.7 ± 0.51^s^	7.3 ± 0.16^r^	0.23	0.17
Neghal									
12.5, 8, 25	n.d.	3.2 ± 0.07^cd^	5.9 ± 0.11d^dc^	3.6 ± 0.10^n^	56.2 ± 0.74^m^	15.6 ± 0.35^e^	8.5 ± 0.26^j^	0.06	0.15
5, 6, 60	n.d.	n.d.	6.3 ± 0.29^cd^	4.8 ± 0.20^c^	66.7 ± 1.28^b^	7.4 ± 0.26^f^	13.3 ± 0.94^g^	0.07	0.20

n.d., not detected, *n* = 3, Data is presented as means ± SD and different letters indicate significant differences (*p* < 0.05).

**Figure 4:**
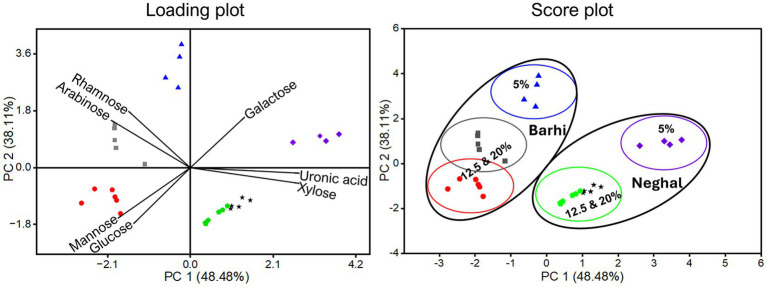
Principal component analysis (loading and score plots) of monosaccharides in Barhi and Neghal hemicelluloses under different conditions (NaOH concentration (5%, 12.5%, 20%), heating time, and temperature).

Principal component analysis (PCA) was used to compare differences in the monosaccharide composition of alkaline hemicellulose extracted under different conditions (alkali concentration, temperature, and time) for Barhi and Neghal cultivars. Figure 4 shows that 86.59% of the total variance was explained by PC1 (48.48%) and PC2 (38.11%), suggesting the feasibility and sufficiency of the PCA (44). PC1 is mainly driven by variations in galactose, xylose and uronic acids (positive loadings) and glucose and mannose (negative loadings), whereas PC2 is driven by rhamnose and arabinose (positive loadings) opposed to glucose and mannose (negative loadings, Supplementary Table S4). Barhi hemicellulose samples cluster primarily at higher PC2 scores and lower and near-zero PC1 scores, indicating greater proportions of rhamnose, arabinose, mannose and glucose, while Neghal hemicellulose samples cluster towards positive PC1 scores and lower PC2 scores, indicating greater xylose, galactose and uronic acids. Mean PC1 scores varied significantly (*p* < 0.05) between cultivars, reflecting the separation shown in Figure 4. Within each cultivar, increasing NaOH concentration (5–20%) shifts samples towards higher glucose and mannose contributions.

### 3.4 Nuclear magnetic resonance spectroscopy

**Figure 5:**
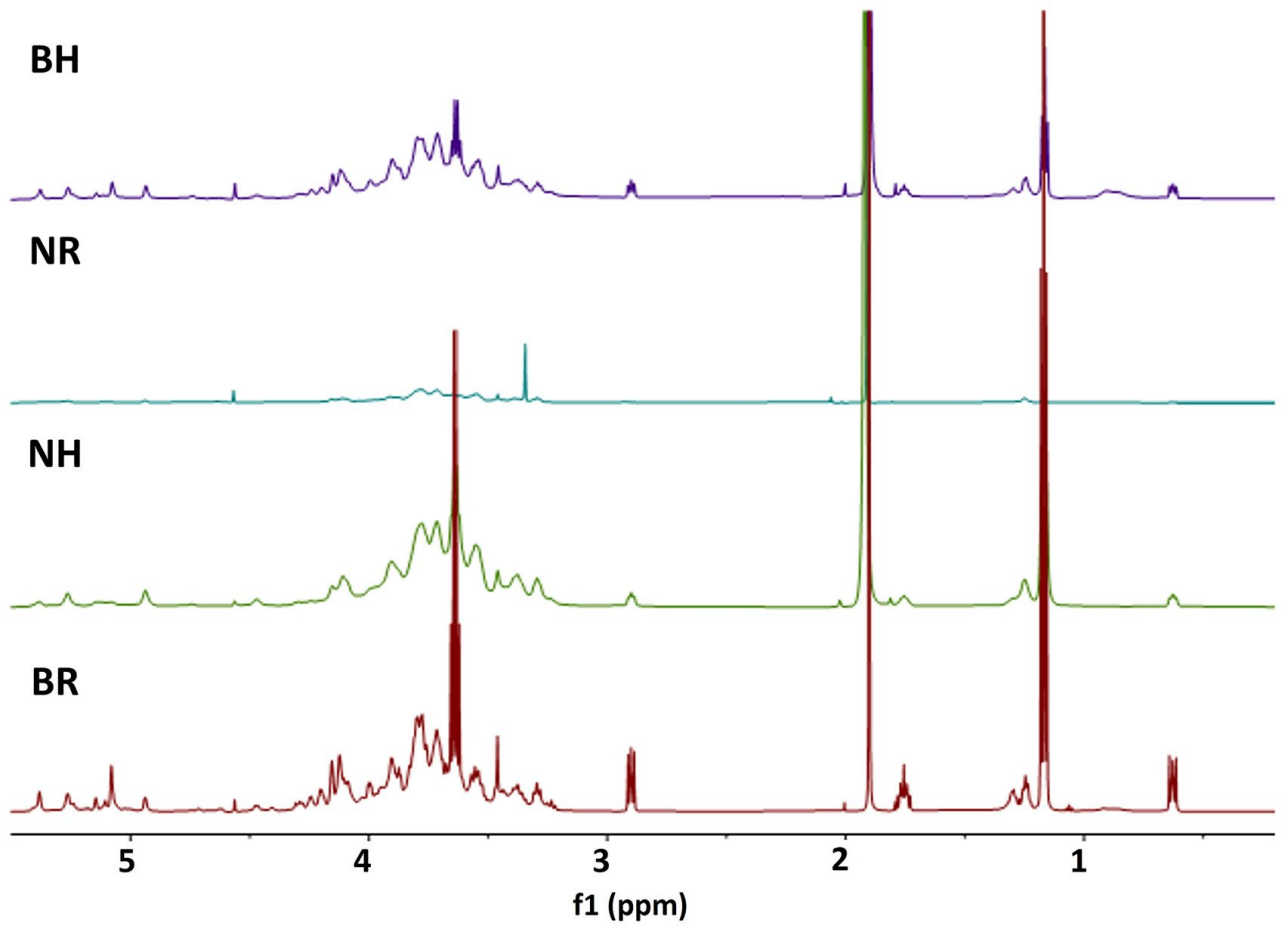
^1^H NMR of the hemicelluloses. BH and BR represent hemicelluloses extracted from Barhi date fruits using alkaline concentration/time/temperature of 5%/6 h/60 °C and 12.5%/8 h/25 °C, respectively, while NH and NR denote hemicelluloses extracted from Neghal date fruits under the same conditions.

**Figure 6:**
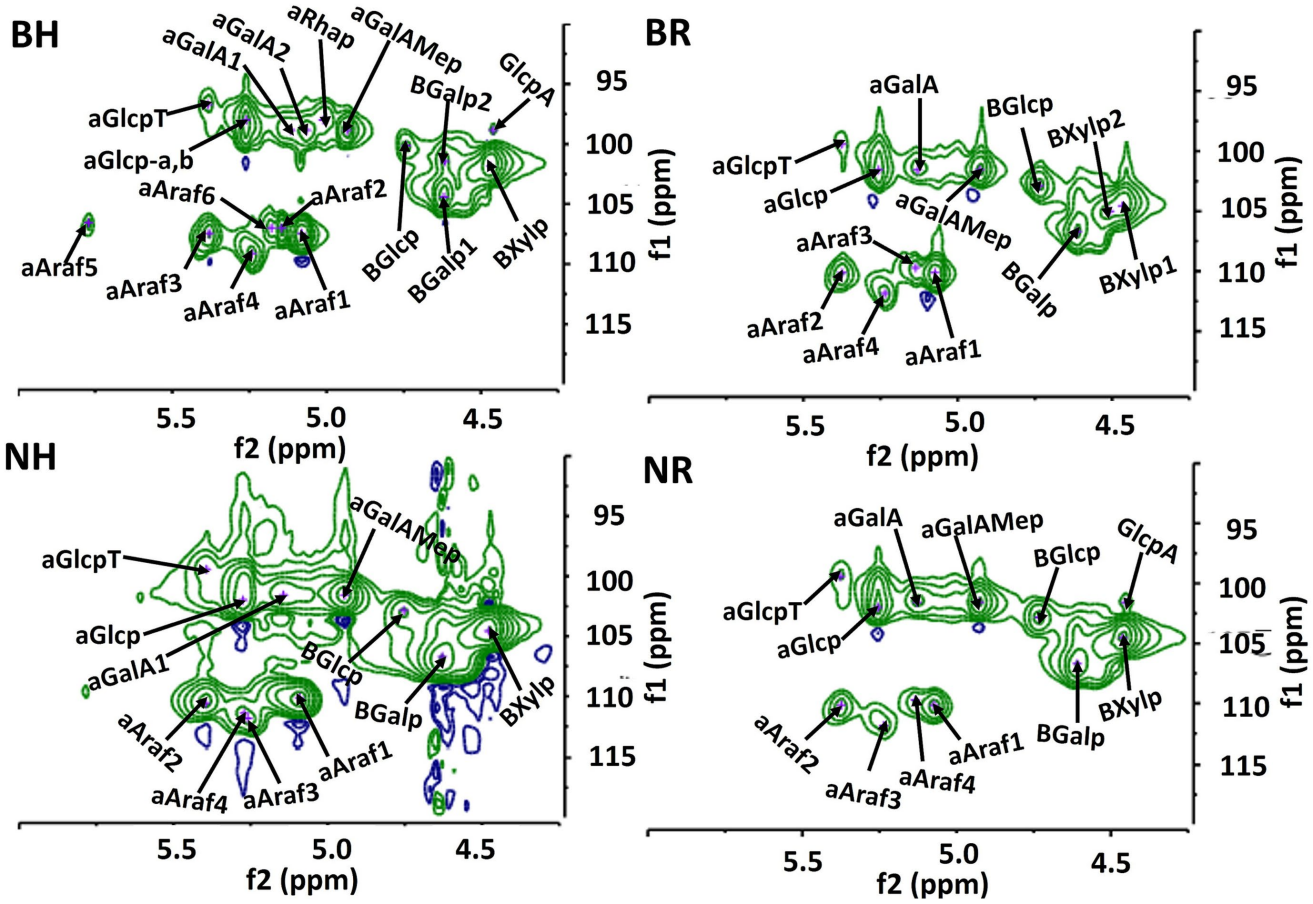
^1^H/^13^C HSQC NMR spectrum of date fruit hemicelluloses. BH and BR indicate hemicelluloses extracted from Barhi date fruits using alkaline concentration/time/temperature of 5%/6 h/60 °C and 12.5%/8 h/25 °C, respectively, while NH and NR denote hemicelluloses extracted from Neghal date fruits under the same conditions.

**Figure 7:**
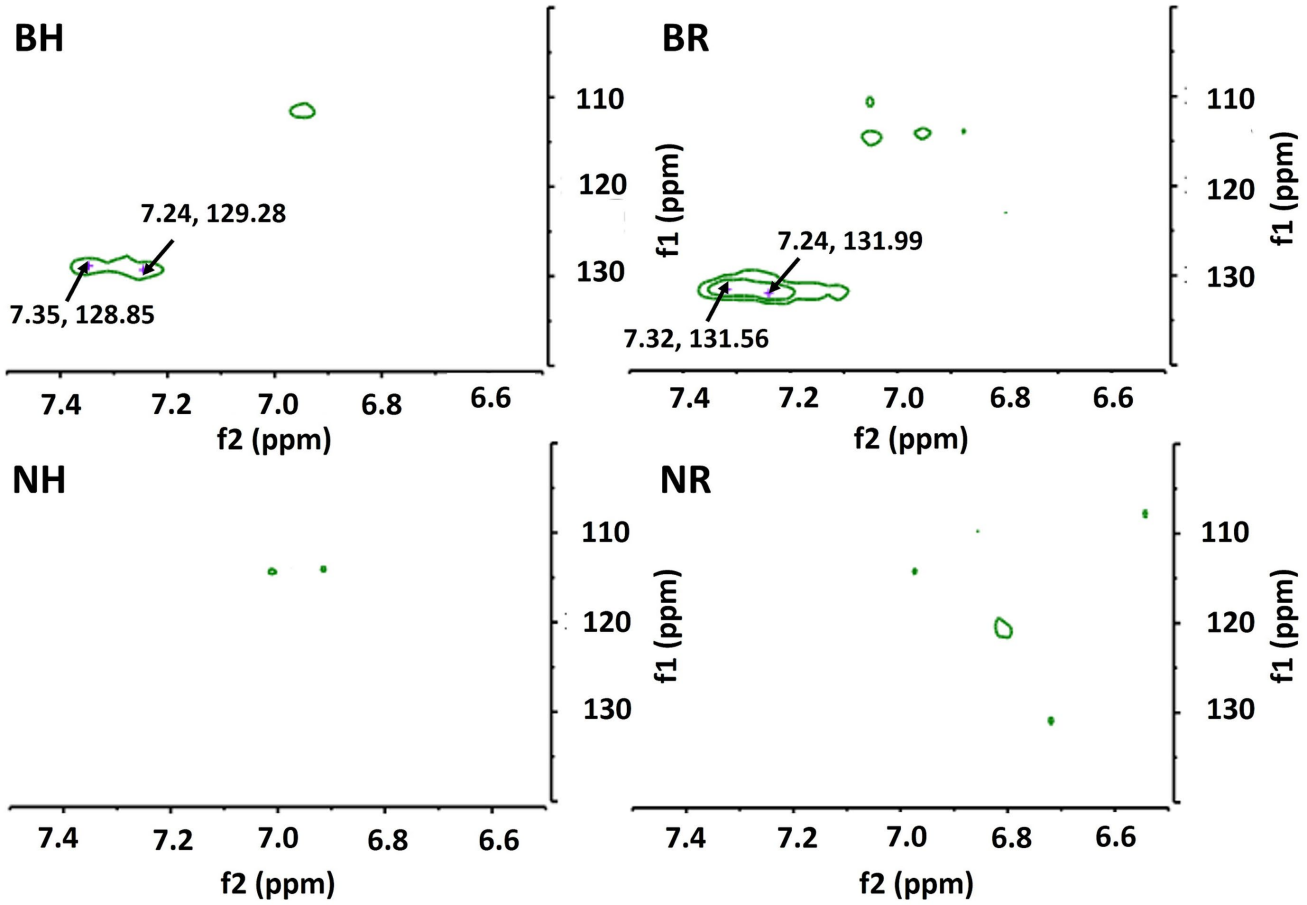
Aromatic rings of the amino acid residues. BH and BR represent hemicelluloses extracted from Barhi date fruits using alkaline concentration/time/temperature of 5%/6 h/60 °C and 12.5%/8 h/25 °C, respectively, while NH and NR are hemicelluloses extracted from Neghal date fruits under the same conditions.

The ^1^H and 2D NMR spectra (Figures 5, 6) confirmed the presence of xylose, glucose, uronic acids, arabinose, galactose and rhamnose in the alkali-extracted date fruit hemicellulose, in agreement with the monosaccharide result (Table 3). The anomeric protons resonate in the *δ* 6.0–4.3 region, whereas signals in the δ 4.5–3.3 region showed heavy overlap, so detailed assignments relied on HSQC, COSY, TOCSY and HMBC experiments (Figure 6; Supplementary Figures S2–S16) together with literature data. The strong anomeric signal at δ 4.47/104.4 in the HSQC spectrum (Figure 6; Table 4) was assigned to *β*-ᴅ-xylopyranose (β-ᴅ-Xylp), and HMBC correlations between H-1 and C-4 of neighboring residues supported a β-(1 → 4)-linked xylan backbone. The downfield signal of C-2 (δ 3.30/74.3) is consistent with O-2-substituted Xylp units, and, together with the dominance of arabinose (1 → 5)-*α*-L-Araf and minor glucuronic acid (1 → 4)-α-ᴅ-GalA and galactose (1 → 3)-β-ᴅ-Galp in the composition data, indicates that the main hemicellulose is arabinoxylans with smaller contributions from glucurono- and galactoarabinoxylan-type motifs. The multiple α-L-arabinofuranose (α-L-Araf) anomeric signals (δ 5.08–5.77/109.1–111.7) and intra-residue HMBC correlations are consistent with (1 → 5)-α-L-arabinan segments and arabinose side chains typical of arabinan/arabinogalactan side chains. Barhi hemicellulose exhibited greater relative intensities for these Araf signals than Neghal (Tables 3; Supplementary Tables S3, S5). Signals for α-ᴅ-GalA and methyl-esterified GalA (GalpA-6-OMe), supported by HMBC correlations to methoxy carbons, indicate that some insoluble pectic homogalacturonan exist in the hemicellulose fractions. HSQC cross-peaks characteristic of (1 → 3)-linked-β-ᴅ-Galp residues (δ 4.62/107.0) together with minor terminal β-ᴅ-Galp signals (δ 4.62/104.0), indicates the presence of Type-II arabinogalactan-like side chains (AG-II). These AG-like features and rhamnose signals consistent with rhamnogalacturonan-I (RG-I) are more pronounced in the Barhi fraction, but due to spectral overlap we restrict our interpretation to the presence of AG/RG-like motifs. The glucose residues (Figure 6; Table 4) are mainly α-ᴅ-glucopyranose (α-ᴅ-Glcp) with minor β-ᴅ-Glcp signals. Anomeric signal at δ 5.3/100–99 and δ 4.74/102.7, together with COSY/TOCSY and HMBC correlations indicate the presence of α-(1 → 3), (1 → 4)-linked glucan in the hemicellulosic fractions. A linear (1 → 3)–(1 → 4)-α-ᴅ-glucan has also been found in fruit from *Mauritia flexuosa* palm, a member of the Arecaceae family, demonstrating its water-insoluble nature and distinct from starch, as it was hydrolyzed by α-amylase and yet does not form a complex with iodine (18). In Barhi hemicellulose, additional aromatic rings of amino acid signals (δ 7–7.5/120–135) were observed (Figure 7), consistent with the presence of protein/peptide component (phenylalanine, tyrosine, and tryptophan). This observation agrees with the measured protein content in the Barhi hemicellulose (4%), which was absent in the Neghal hemicellulose. The protein content in Barhi hemicellulose was within the range reported for AGP-containing cell wall fractions (protein: 1%–10%) (45–48). Additionally, the methylene and methyl protons of amino acid residues (e.g., alanines, leucines, valines, and isoleucines) exist in the regions of 1.5 to 3.5 ppm and 0 to 1.4 ppm (Figure 5) and the polypeptide core (hydroxyproline, alanine, serine, or threonine) signals exist in the region of 6 to 10 ppm in the HSQC spectrum (Supplementary Figures S6, S10, S12, S16).

**Table 4:** Identified HSQC, COSY, and TOCSY NMR chemical shifts.

Residues	1H/1C	2H/2C	3H/3C	4H/4C	5H/5C	6H/6C
β-ᴅ-Xylp-1	4.47/104.4	3.30/74.3	3.54/76.6	4.10/79.1	3.77/63.7	n.d.
α-ᴅ-Glcp-a,b	5.26/100.5	3.54/74	n.d.	4.30/81.7	3.76/75.7	3.64/60.3
β-ᴅ-Glcp	4.74/102.7	n.d.	4.25/87.3	n.d.	n.d.	n.d.
α-ᴅ-Glcp-T	5.37/99.3	n.d.	n.d.	n.d.	n.d.	n.d.
α-ᴅ-GalA-1	5.07/101.4	n.d.	n.d.	n.d.	n.d.	n.d.
α-ᴅ-GalA-2	5.12/101.4	3.65/82.55	n.d.	n.d.	n.d.	n.d.
α-ᴅ-GalAMep	4.94/101.4	n.d.	n.d.	n.d.	n.d.	n.d.
α-ᴅ-GlcpA	4.46/101.4	n.d.	n.d.	n.d.	n.d.	n.d.
α-L-Araf-1	5.08/110.0	4.11/84.7	n.d.	3.99/79.6	3.88/69.7	n.d.
α-L-Araf-2	5.15/106.6	4.09/86.6	n.d.	n.d.	n.d.	n.d.
α-L-Araf-3^a,b^	5.38/110.0	4.19/80	n.d.	n.d.	n.d.	n.d.
α-L-Araf-4	5.24/111.7	4.13/83.4	n.d.	n.d.	n.d.	n.d.
α-L-Araf-5	5.77/109.1	n.d.	n.d.	n.d.	n.d.	n.d.
α-L-Araf-6	5.18/109.5	4.29/84.3	n.d.	n.d.	n.d.	n.d.
β-ᴅ-Galp-1	4.62/107.0	n.d.	n.d.	n.d.	n.d.	4.10/65.4
β-ᴅ-Galp-2	4.62/103.4	n.d.	n.d.	n.d.	n.d.	n.d.
α-L-Rhap-1	5.01/100.6	n.d.	n.d.	n.d.	3.78/70.3	1.24/19.1
α-L-Rhap-2	5.01/100.6	n.d.	n.d.	n.d.	3.85/70.5	1.30/19.6

n.d., not detected, GalAMep represents methyl-esterified galacturonic acid residue; GlcpA represent glucuronic acid residue; 1, 2, 3, represent different environments, ^a,b^: labels for two distinct residues whose anomeric signals overlap.

Overall, the compositional and NMR data indicate that both fractions are complex alkali-soluble assemblies dominated by β-(1 → 4)-xylan backbones with Araf- and uronic acid-containing side chains, together with minor glucan and pectic components. Barhi hemicellulose showed relatively stronger arabinogalactan (AG)/arabinogalactan-protein (AGP)-, and RG-I-like features than Neghal, whereas Neghal hemicellulose showed less branched, more arabinoxylan-dominated profile. There is a possible covalent interaction between xylan, glucan, pectin and AGP, as reported in related systems (45, 47–49, 60, 61), however, our HMBC spectra does not adequately define such network in date hemicellulose; thereby requiring further studies. Also, the branched arabinoxylan- and AGP/pectin-related motifs in the Barhi are consistent with its softer texture. This is in line with previous work which established a correlation between hardness of dates with higher proportions of lignin, arabinoxylan and pectin (25). Studies in other fruits have associated AGP–pectin associations and Ca^2+^-linked pectin networks in the control of tissue hardness and softening (50–53). While similar mechanisms may operate in date fruits, additional targeted experiments would be required to establish a direct causal link between these structural features and fruit softening.

### 3.5 Molecular weight analysis

**Figure 8:**
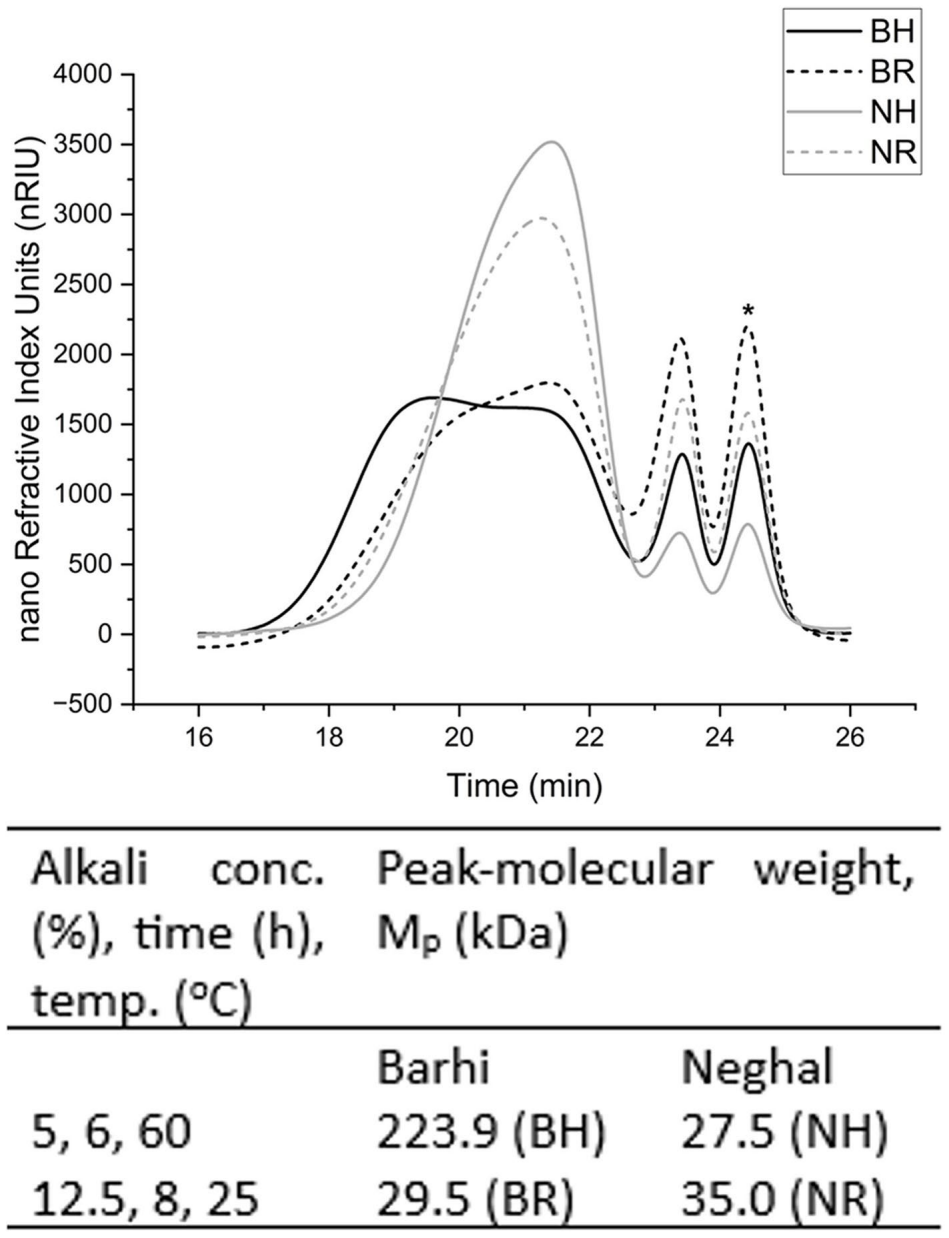
Molecular weight distribution and peak molecular weight of hemicelluloses from Barhi and Neghal cultivars. Barhi, 5% NaOH, 6 h, 60 °C (BH, solid black); Barhi, 12.5% NaOH, 8 h, 25 °C (BR, dashed black); Neghal, 5% NaOH, 6 h, 60 °C (NH, solid grey); Neghal, 12.5% NaOH, 8 h, 25 °C (NR, dashed grey). The peak marked “*solvent peak” at 24.5 min corresponds to the elution of the injection solvent (NaNO_3_) and was not used for molecular weight determination.

HPSEC analysis revealed that Barhi hemicellulose had a broader elution profile than Neghal hemicellulose, indicating a more heterogeneous polymer (Figure 8). The pullulan-equivalent molecular weight (M_p_) of Barhi hemicellulose (29.5 to 223.9 kDa) was considerably higher than that of Neghal hemicellulose (27.5–35.0 kDa). The molecular weights of hemicellulose from other fruits includes *Astrocaryum aculeatum* fruit (24–119 kDa) (20), and *Nelumbo nucifera* Gaetn fruit (108–117 kDa) (54). For Neghal, increasing the alkali concentration (5%–12.5%) and heating time (6–8 h) while reducing temperature (60 °C–25 °C) resulted in a slight increase in M_p_ (27.5 kDa to 35.0 kDa), Conversely, under the same change in conditions the Barhi fraction shifted from a high M_p_ (223.9 kDa) to a lower M_p_ (29.5 kDa), suggesting that harsher extraction conditions may partly degrade or fragment high-molecular-weight assemblies in Barhi. These configurations likely reflect both inherent structural differences between the cultivars (Figures 6, 7) and the different sensitivity of their cell wall to alkaline solubilization and degradation, for instance between less substituted arabinoxylan in Neghal and more branched or pectin-associated polymers in Barhi (55). Other factors (e.g., degree of branching, aggregation, covalently linked multi-domain structures, degree of polymerization, etc.) may also contribute to the high peak-molecular weight in Barhi hemicellulose.

### 3.6 Scanning electron and optical microscopy

**Figure 9:**
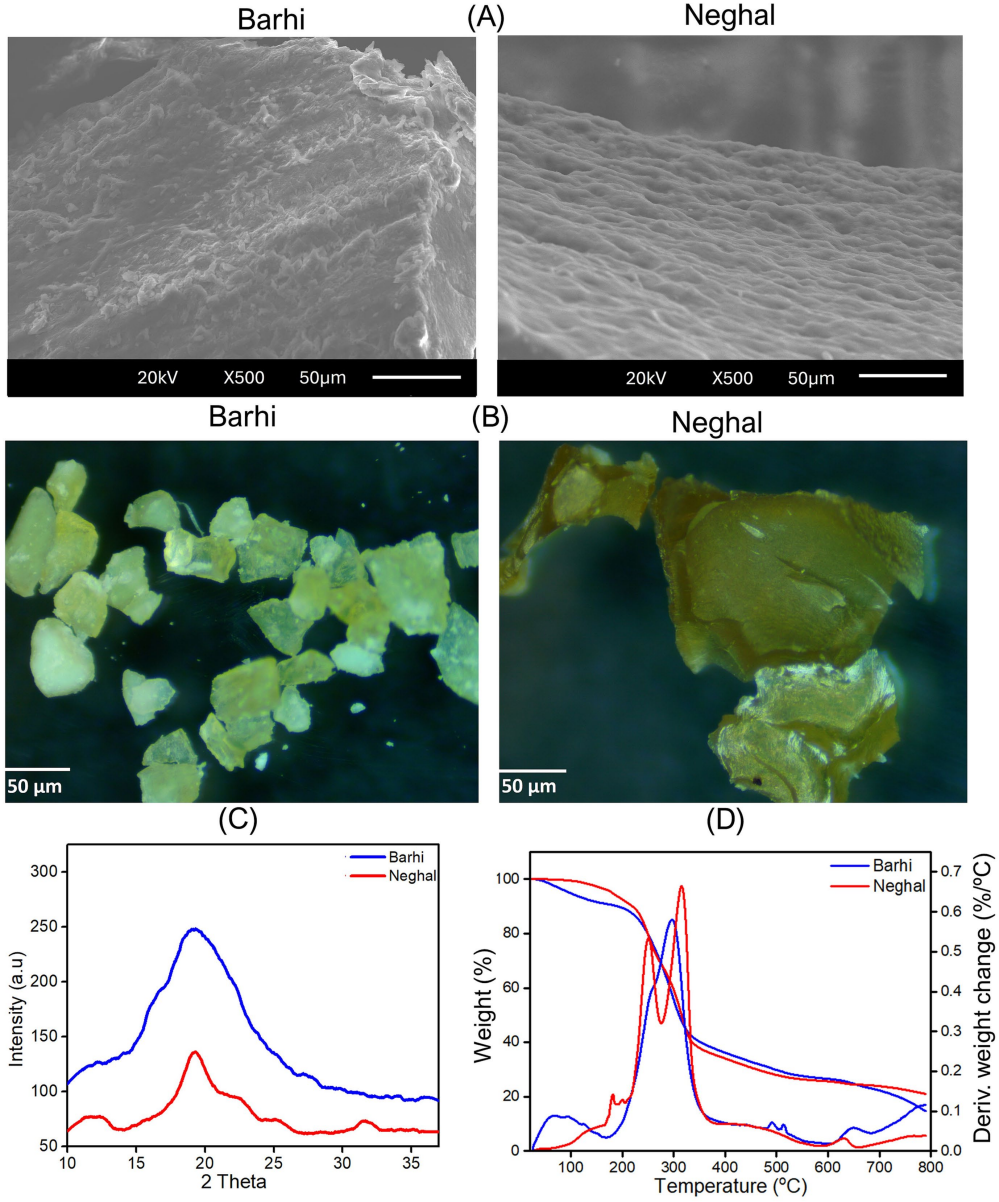
Scanning electron microscopy **(A)**, optical microscopy **(B)**, X-ray diffraction spectroscopy **(C)**, derivative thermogravimetric and thermogravimetric analysis **(D)** of hemicellulose fractions (Barhi and Neghal cultivars). Samples analyzed were hemicelluloses obtained from Barhi and Neghal date fruits using alkali concentration/time/temperature of 5%/6 h/60 °C.

The Barhi hemicellulose fraction exhibited small, irregular, more fibrillar, and fragmented particles, while Neghal hemicellulose fraction displayed larger, sheet-like aggregates, with smoother and denser surfaces (Figures 9A,B). These qualitative differences are consistent with the higher xylose content and lower branching seen in the Neghal hemicellulose (Table 3; Figure 4), which may support closer chain packing and aggregation (56). Similar irregular-sized particles have been reported for hemicellulose from oil palm empty fruit bunch (57) and pineapple peel waste (58).

### 3.7 X-ray diffraction

The X-ray diffraction (XRD) curve (Figure 9C) of hemicellulose exhibited two broad maxima, one near 12° and another around 19°. The broad nature of these peaks indicates that the isolated hemicellulose is predominantly amorphous with only limited short-range order (semi-crystalline domains). The Barhi fraction demonstrated broader and less pronounced maximum near 19° than Neghal, suggesting a more disordered packing in the alkali-soluble hemicellulose in Barhi. Xylan-type hemicellulose is generally regarded as amorphous, although non-substituted xylan segments can exhibit both hydrous and anhydrous ordered structures, while branching and acetylation tend to impede crystallization (22). Amorphous xylan-type hemicellulose is useful for producing film and coating materials, while crystalline xylan-type hemicellulose is suitable for emulsifiers and dispersants.

### 3.8 Thermal properties

Thermogravimetric analysis was used to analyze the thermal stability of the alkali-soluble hemicellulose fractions. The hemicellulose curve (Figure 9D) shows an initial peak (0 °C–180 °C) corresponding to evaporation of moisture and loosely bound volatiles, a main peak (200 °C–380 °C) corresponding to the decomposition of the hemicellulosic polysaccharides, and small peak (600 °C–700 °C) ascribed to char formation (24, 54). Both Barhi and Neghal hemicellulosic fractions had comparable main decomposition (200 °C–380 °C), however, within this region, Neghal hemicellulose showed noticeable splitting peaks, while Barhi hemicellulose showed broad peak. The broad decomposition peak (200 °C–380 °C) in the Barhi hemicellulosic fraction can be correlated to the broad molecular weight distribution (Figure 8), structural complexity of the polysaccharides in Barhi fraction, and crystalline and amorphous regions (54), whereas, the peak splitting in the main decomposition region (200 °C–380 °C) of the Neghal hemicellulose was attributed to the removal of the uronic acid and arabinose branches from the main chain and degradation of small molecular mass components in the hemicellulosic fraction (59).

## 4 Conclusion

This study investigated the composition and properties of alkali-extracted hemicellulose from two date fruit cultivars, Barhi (soft) and Neghal (hard). The hemicellulosic fractions were predominantly composed of arabinoxylan with a small portion of glucurono- and galacto-arabinoxylan motifs. It also contains insoluble glucan, pectic homogalacturonan (HG), and rhamnogalacturonan-I (RG-I) components. Neghal hemicellulose contained relatively less branched arabinoxylan and had lower molecular weight (27.5–35 kDa), whereas Barhi hemicellulose was enriched in arabinogalactan protein (AGP) and RG–like signatures, and exhibited higher molecular weight (29.5–223.9 kDa). These structural features are consistent with a more branched, hydrated and less tightly packed hemicellulose–pectin matrix in Barhi, and a more arabinoxylan-dominated and relatively more ordered matrix in Neghal. Together with other factors (e.g., sucrose versus invert sugars and soluble pectin), these differences may contribute to the softer texture of Barhi and the greater hardness of Neghal. However, more targeted mechanical and biochemical studies, using more date fruit varieties, will be required to establish and quantify these direct links. Overall, our results contribute to the existing knowledge of hemicellulosic polysaccharides in the cell walls of date fruits. Further studies can focus on the fractionation and quantification of individual hemicellulosic components and their interactions with other dietary-fiber constituents across additional cultivars to provide more structural and functional insight.

## Data Availability

The original contributions presented in the study are included in the article/Supplementary material, further inquiries can be directed to the corresponding author.
