# Supplementary material for: Chemical and structural characterization of hemicellulose from date fruits (Phoenix dactylifera L.)
Source: Front Nutr. 2026 Mar 26;13:1804879. doi: 10.3389/fnut.2026.1804879 (PMC13062190; doi:10.3389/fnut.2026.1804879)
Supplement: Supplementary file 1 [file Supplementary_file_1.docx]

***Supplementary Material***

**Table S1.** ANOVA for the regression models of hemicellulose yield selected

| Source | Sum of square | Degree of freedom | Mean square | F-value | p>F |
| --- | --- | --- | --- | --- | --- |
| Barhi | | | | | |
| Model | 418.26 | 3.00 | 139.42 | 9.46 | <0.01 |
| A-alkaline concentration | 375.91 | 1.00 | 375.91 | 25.50 | <0.01 |
| B-heating time | 0.5781 | 1.00 | 0.5781 | 0.04 | 0.85 |
| C-temperature | 41.77 | 1.00 | 41.77 | 2.83 | 0.12 |
| Residual | 162.16 | 11.00 | 14.74 | - | - |
| Lack of fit | 156.76 | 9.00 | 17.42 | 0.46 | 0.14 |
| Pure Error | 5.40 | 2.00 | 2.70 | - | - |
| Cor Total | 580.41 | 14.00 | - | - | - |
| Neghal | | | | | |
| Model | 297.68 | 3.00 | 99.23 | 6.46 | <0.01 |
| A-alkaline concentration | 293.91 | 1.00 | 293.91 | 19.13 | <0.01 |
| B-heating time | 2.90 | 1.00 | 2.90 | 0.19 | 0.67 |
| C-temperature | 0.87 | 1.00 | 0.87 | 0.06 | 0.82 |
| Residual | 168.96 | 11.00 | 15.36 | - | - |
| Lack of fit | 168.91 | 9.00 | 18.77 | 0.53 | 0.19 |
| Pure Error | 0.06 | 2.00 | 0.03 | - | - |
| Cor Total | 466.64 | 14.00 | - | - | - |

**Table. S2**. Model selection criteria for hemicellulose yield for Barhi and Neghal cultivars

|  | Linear | 2FI | Quadratic | Cubic |
| --- | --- | --- | --- | --- |
| Barhi | | | | |
| Sequential p-value | 0.0022 | 0.9753 | 0.0834 | 0.1701 |
| Lack of fit p-value | 0.1413 | 0.0989 | 0.1701 | - |
| Adjusted R^2^ | 0.6444 | 0.5233 | 0.7774 | 0.9349 |
| Predicted R^2^ | 0.5402 | 0.1318 | 0.1444 | - |
| Neghal | | | | |
| Sequential p-value | 0.0088 | 0.7582 | 0.0532 | 0.0023 |
| Lack of fit p-value | 0.0015 | 0.0011 | 0.0023 | - |
| Adjusted R^2^ | 0.5392 | 0.4487 | 0.7767 | 0.9992 |
| Predicted R^2^ | 0.3242 | -0.2588 | -0.2173 | - |

**Table S3.** Monosaccharides composition of alkaline hemicelluloses and DMSO hemicelluloses

| Alkali conc. (%), time (h), temp. (^o^C) | Rhamnose (%) | Mannose (%) | Galactose (%) | Arabinose (%) | Xylose (%) | Glucose (%) | Uronic acid (%) | Arabinose/Xylose ratio | Uronic acid/Xylose ratio |
| --- | --- | --- | --- | --- | --- | --- | --- | --- | --- |
| Barhi alkaline hemicellulose | | | | | | | | | |
| 20, 6, 60 | 2.52±0.02^a^ | 3.19±0.09^b^ | 6.14±0.11^c^ | 9.85±0.13^d^ | 40.58±0.23^e^ | 12.57±0.48^f^ | 6.43±0.13^c^ | 0.24 | 0.16 |
| 12.5, 4, 25 | 0.57±0.22^b^ | 3.63±0.15^cb^ | 4.77±0.04^a^ | 8.58±0.16^e^ | 33.08±0.17^f^ | 13.49±0.04^i^ | 5.47±0.16^g^ | 0.25 | 0.17 |
| 5, 4, 42.5 | 2.55±0.12^a^ | n.d. | 5.92±0.26^c^ | 9.09±0.47^h^ | 38.38±0.54^e^ | 7.63±0.02^j^ | 7.02±0.52^c^ | 0.24 | 0.18 |
| 20, 8, 42.5 | 1.61±0.94^k^ | 3.33±0.32^cb^ | 6.10±0.77^c^ | 9.81±1.26^d^ | 36.27±1.83^e^ | 12.99±1.91^l^ | 6.58±1.27^c^ | 0.27 | 0.18 |
| 5, 6, 25 | 2.96±0.18^a^ | n.d. | 6.78±0.53^m^ | 9.76±0.77^d^ | 45.26±1.13^n^ | 9.78±0.93^d^ | 7.00±0.33^c^ | 0.22 | 0.15 |
| 12.5, 8, 60 | 2.47±0.16^a^ | 2.76±0.26^ac^ | 6.05±0.36^c^ | 8.89±0.69^he^ | 40.73±1.16^e^ | 12.60±0.76^f^ | 5.60±0.28^co^ | 0.22 | 0.13 |
| 12.5, 6, 42.5 | 0.70±0.79^b^ | 3.28±0.02^cb^ | 5.46±0.29^c^ | 7.03±0.17^p^ | 37.44±0.92^e^ | 15.89±0.47^k^ | 6.43±0.16^c^ | 0.19 | 0.17 |
| 12.5, 6, 42.5 | 1.40±0.78^k^ | 3.30±0.31^cb^ | 5.51±0.25^c^ | 5.84±0.21^c^ | 36.88±1.64^e^ | 14.85±0.79^kb^ | 5.64±0.37^co^ | 0.16 | 0.15 |
| 5, 8, 42.5 | 2.53±0.06^a^ | n.d. | 5.98±0.12^c^ | 7.70±0.49^q^ | 39.93±0.47^e^ | 8.31±0.12^o^ | 7.28±0.44^r^ | 0.19 | 0.18 |
| 12.5, 6, 42.5 | n.d. | 3.51±0.06^cb^ | 5.85±0.20^c^ | 9.10±0.73^h^ | 40.53±0.68^e^ | 17.16±0.48^k^ | 6.15±0.14^cd^ | 0.22 | 0.15 |
| 12.5, 8, 25 | n.d. | 3.23±0.75^cb^ | 5.08±0.33^ac^ | 6.47±0.37^b^ | 36.99±0.68e | 14.13±0.97^kb^ | 6.03±0.34^cd^ | 0.17 | 0.16 |
| 5, 6, 60 | 3.13±0.16^c^ | n.d. | 6.51±0.29^s^ | 10.13±1.52^d^ | 43.62±1.36^n^ | 6.70±0.51^s^ | 7.30±0.16^r^ | 0.23 | 0.17 |
| 12.5, 4, 60 | 2.40±0.10^a^ | 2.91±0.05^ac^ | 6.27±0.20^c^ | 7.69±0.76^q^ | 40.03±1.49^e^ | 15.95±0.53^k^ | 5.95±0.59^co^ | 0.19 | 0.15 |
| 20, 6, 25 | 2.14±0.12^h^ | 3.56±0.21^cb^ | 5.90±0.32^c^ | 6.40±0.46^b^ | 34.95±1.94^e^ | 13.71±0.82^i^ | 9.08±0.21^fg^ | 0.18 | 0.26 |
| 20, 4, 42.5 | 1.52±0.86^k^ | 3.18±0.25^b^ | 5.31±0.14^ca^ | 7.59±0.66^q^ | 34.74±1.82^ef^ | 15.89±0.79^k^ | 6.08±0.23^cd^ | 0.22 | 0.18 |
| Neghal alkaline hemicellulose | | | | | | | | | |
| 20, 6, 60 | n.d. | 2.06±0.12^f^ | 5.68±0.10^d^ | 4.64±0.10^c^ | 61.09±0.78^f^ | 15.24±0.28^e^ | 8.99±0.47^j^ | 0.08 | 0.15 |
| 12.5, 4, 25 | n.d. | 3.07±0.05^c^ | 5.61±0.19^d^ | 4.20±0.13^c^ | 60.21±1.89^g^ | 15.34±0.51^e^ | 8.39±0.45^j^ | 0.07 | 0.14 |
| 5, 4, 42.5 | n.d. | n.d. | 6.55±0.33^dc^ | 4.70±0.29^cd^ | 68.28±1.16^b^ | 9.04±0.47^m^ | 11.30±0.66^n^ | 0.07 | 0.17 |
| 20, 8, 42.5 | n.d. | 2.69±0.06^ac^ | 5.66±0.29^d^ | 3.31±0.15^n^ | 54.44±0.64^m^ | 13.45±0.30^en^ | 8.91±0.19^j^ | 0.06 | 0.16 |
| 5, 6, 25 | n.d. | n.d. | 6.70±0.22^m^ | 4.12±0.17^c^ | 70.62±0.79^b^ | 10.13±0.15^d^ | 11.25±0.45^n^ | 0.06 | 0.16 |
| 12.5, 8, 60 | n.d. | 1.58±0.04^g^ | 5.50±0.49^d^ | 3.41±0.13^n^ | 60.07±1.86^f^ | 13.73±0.91^ef^ | 8.63±0.28^j^ | 0.06 | 0.14 |
| 12.5, 6, 42.5 | n.d. | 2.53±0.04^ca^ | 6.06±0.07^dc^ | 3.71±0.25^n^ | 63.80±0.04^fb^ | 13.42±0.07^en^ | 9.68±0.27^t^ | 0.06 | 0.15 |
| 12.5, 6, 42.5 | n.d. | 2.41±0.18^ca^ | 5.65±0.20^d^ | 3.21±0.14^n^ | 59.66±1.29^f^ | 12.39±0.27^o^ | 9.35±0.08^t^ | 0.05 | 0.16 |
| 5, 8, 42.5 | n.d. | n.d. | 6.23±0.31^cd^ | 4.51±0.14^c^ | 66.16±1.33^b^ | 8.66±0.24^t^ | 9.49±0.79^t^ | 0.07 | 0.14 |
| 12.5, 6, 42.5 | n.d. | 2.75±0.24^ac^ | 5.92±0.29^dc^ | 3.72±0.16^n^ | 61.36±1.92^f^ | 13.61±0.79^en^ | 8.85±0.27^j^ | 0.06 | 0.14 |
| 12.5, 8, 25 | n.d. | 3.19±0.07^cd^ | 5.88±0.11^dc^ | 3.59±0.10^n^ | 56.20±0.74^m^ | 15.57±0.35^e^ | 8.52±0.26^j^ | 0.06 | 0.15 |
| 5, 6, 60 | n.d. | n.d. | 6.33±0.29^cd^ | 4.83±0.20^c^ | 66.71±1.28^b^ | 7.37±0.26^f^ | 13.32±0.94^g^ | 0.07 | 0.20 |
| 12.5, 4, 60 | n.d. | 2.06±0.06^f^ | 5.72±0.15^d^ | 3.95±0.18^nc^ | 60.92±1.90^f^ | 12.31±0.61^o^ | 9.40±0.63^t^ | 0.06 | 0.15 |
| 20, 6, 25 | n.d. | 3.12±0.29^c^ | 5.50±0.06^d^ | 3.47±0.01^cn^ | 52.81±1.36^r^ | 14.07±0.51^en^ | 8.92±0.24^j^ | 0.07 | 0.17 |
| 20, 4, 42.5 | n.d. | 2.65±0.20^ac^ | 5.83±0.58^dc^ | 3.33±0.41^n^ | 57.39±1.06^fm^ | 13.16±0.47^l^ | 8.79±0.23^j^ | 0.06 | 0.15 |
| DMSO hemicellulose | | | | | | | | | |
| Barhi | n.d. | n.d. | 7.05±0.20^cd^ | 6.03±0.24^bd^ | 24.76±0.11^n^ | 17.20±0.35^f^ | 6.32±0.44^cd^ | 0.24 | 0.25 |
| Neghal | 1.27^ab^ | n.d. | 13.38±0.10^e^ | 6.96±0.33^bd^ | 21.74±0.43^n^ | 14.78±0.56^e^ | 7.41±0.22^d^ | 0.32 | 0.34 |

n=3. Data is presented as means ± SD and different letters indicate significant differences in each column (p < 0.05).

**Table S4.** Loading matrix

| Variables | PC1 loading | PC2 loading |
| --- | --- | --- |
| Rhamnose | -0.29 | 0.48 |
| Mannose | -0.34 | -0.43 |
| Galactose | 0.26 | 0.43 |
| Arabinose | -0.37 | 0.40 |
| Xylose | 0.51 | -0.13 |
| Glucose | -0.27 | -0.47 |
| Uronic acids | 0.51 | -0.05 |

**Table S5**. Relative proportions of the sugar residues from HSQC anomeric region

| Residues | Volume | Percentage (%) | Total residues (%) |  |
| --- | --- | --- | --- | --- |
| NH | | | | |
| H1-C1 α-ᴅ-Glcp | 17869.66 | 11.38 | Total α-L-Araf | 13.34 |
| H1-C1 α-ᴅ-Glcp-T | 2286.45 | 1.46 | Total α-ᴅ-GalpA | 18.89 |
| H1-C1 α-L-Araf-1 | 10152.59 | 6.46 | Total α-L-Rhap | - |
| H1-C1 α-L-Araf-2 | 4236.84 | 2.70 | Total β-ᴅ-Glcp | 2.65 |
| H1-C1 α-L-Araf-3 | 3101.72 | 1.98 | Total β-ᴅ-Xylp | 31.55 |
| H1-C1 α-L-Araf-4 | 3463.11 | 2.21 | Total β-ᴅ-Galp | 20.73 |
| H1-C1 α-ᴅ-GalpAMep | 16274.83 | 10.36 | Total α-ᴅ-Glcp | 12.83 |
| H1-C1 β-ᴅ-Xylp | 49552.78 | 31.55 |  |  |
| H1-C1 β-ᴅ-Glcp | 4157.44 | 2.65 |  |  |
| H1-C1 β-ᴅ-Galp | 32553.8 | 20.73 |  |  |
| H1-C1 α-ᴅ-GalpA | 13393.61 | 8.53 |  |  |
| BH | | | | |
| H1-C1 α-L-Araf-6 | 569.61 | 1.31 | Total α-L-Araf | 28.60 |
| H1-C1 α-L-Araf-5 | 189.05 | 0.44 | Total α-ᴅ-GalpA | 16.12 |
| H1-C1 α-L--Araf-4 | 1367.51 | 3.15 | Total α-L-Rhap | 4.19 |
| H1-C1 α-L-Araf-3 | 1696.33 | 3.91 | Total α-ᴅ-Glcp | 10.09 |
| H1-C1 α-L-Araf-2 | 1427.56 | 3.29 | Total β-ᴅ-Xylp | 20.11 |
| H1-C1 α-L-Araf-1 | 7146.98 | 16.49 | Total β-ᴅ-Galp | 19.09 |
| H1-C1 α-ᴅ-GalpAMep | 2657.4 | 6.13 | Total β-ᴅ-Glcp | 1.51 |
| H1-C1 α-L-Rhap | 1815.87 | 4.19 | Total α-ᴅ-GlcpA | 0.28 |
| H1-C1 α-ᴅ-Glcp-T | 574.73 | 1.33 | Total α-ᴅ-Glcp | - |
| H1-C1 α-ᴅ-Glcp-1,2 | 3800.76 | 8.77 |  |  |
| H1-C1 α-ᴅ-GlcpA | 121.27 | 0.28 |  |  |
| H1-C1 α-ᴅ-GalpA | 1593.02 | 3.68 |  |  |
| H1-C1 α-ᴅ-GalpA | 2737.2 | 6.32 |  |  |
| H1-C1 β-ᴅ-Xylp | 8716.47 | 20.11 |  |  |
| H1-C1 β-ᴅ-Glcp | 654.98 | 1.51 |  |  |
| H1-C1 β-ᴅ-Galp-2 | 2024.85 | 4.67 |  |  |
| H1-C1 β-ᴅ-Galp-1 | 6250.7 | 14.42 |  |  |
| NR | | | | |
| H1-C1 α-L-Araf-4 | 510.75 | 1.44 | Total α-L-Araf | 6.84 |
| H1-C1 α-L-Araf-3 | 776.87 | 2.18 | Total α-ᴅ-GalpA | 6.05 |
| H1-C1 α-L-Araf-2 | 922.9 | 2.59 | Total α-L-Rhap |  |
| H1-C1 α-L-Araf-1 | 1588.78 | 34.04 | Total α-ᴅ-Glcp | 14.16 |
| H1-C1 α-ᴅ-GlcpA | 221.53 | 0.62 | Total β-ᴅ-Xylp | 30.96 |
| H1-C1 α-ᴅ-GalpA | 2150.94 | 6.05 | Total β-ᴅ-Galp | 19.74 |
| H1-C1 β-ᴅ-Xylp | 11013.52 | 30.96 | Total β-ᴅ-Glcp | 4.67 |
| H1-C1 β-ᴅ-Glcp | 1660.9 | 4.67 | Total α-ᴅ-GlcpA | 0.62 |
| H1-C1 β-ᴅ-Galp | 7023.58 | 19.74 |  |  |
| H1-C1 α-ᴅ-GalpAMep | 4667.69 | 13.12 |  |  |
| H1-C1 α-ᴅ-Glcp-T | 516.48 | 1.45 |  |  |
| H1-C1 α-ᴅ-Glcp | 4522.39 | 12.71 |  |  |
| BR | | | | |
| H1-C1 β-ᴅ-Galp | 3704.07 | 11.55 | Total α-L-Araf | 21.37 |
| H1-C1 β-ᴅ-Glcp | 1396.87 | 4.36 | Total α-ᴅ-GalpA | 10.53 |
| H1-C1 β-ᴅ-Xylp-1 | 8195.32 | 25.56 | Total α-L-Rhap | 0 |
| H1-C1 β-ᴅ-Xylp-2 | 5634.64 | 17.57 | Total α-ᴅ-Glcp | 9.06 |
| H1-C1 α-ᴅ-GalpA | 809.09 | 2.52 | Total β-ᴅ-Xylp | 43.14 |
| H1-C1 α-ᴅ-Glcp | 2546.31 | 7.94 | Total β-ᴅ-Galp | 11.55 |
| H1-C1 α-ᴅ-Glcp-T | 358.93 | 1.12 | Total β-ᴅ-Glcp | 4.36 |
| H1-C1 α-ᴅ-GalpAMep | 2565.44 | 8.00 | Total α-ᴅ-GlcpA |  |
| H1-C1 α-L-Araf-1 | 3615.11 | 11.28 |  |  |
| H1-C1 α-L-Araf-2 | 1087.57 | 3.39 |  |  |
| H1-C1 α-L-Araf-3 | 1226.2 | 3.82 |  |  |
| H1-C1 α-L-Araf-4 | 921.65 | 2.87 |  |  |

BH and BR are hemicellulose extracted from Barhi date fruits using alkaline concentration/time/temperature of 5%/6 h/60^o^C and 12.5%/8 h/25^o^C, respectively and NH and NR are hemicellulose extracted from Neghal date fruits using alkaline concentration/time/temperature of 5%/6 h/60^o^C and 12.5%/8 h/25^o^C, respectively.

**
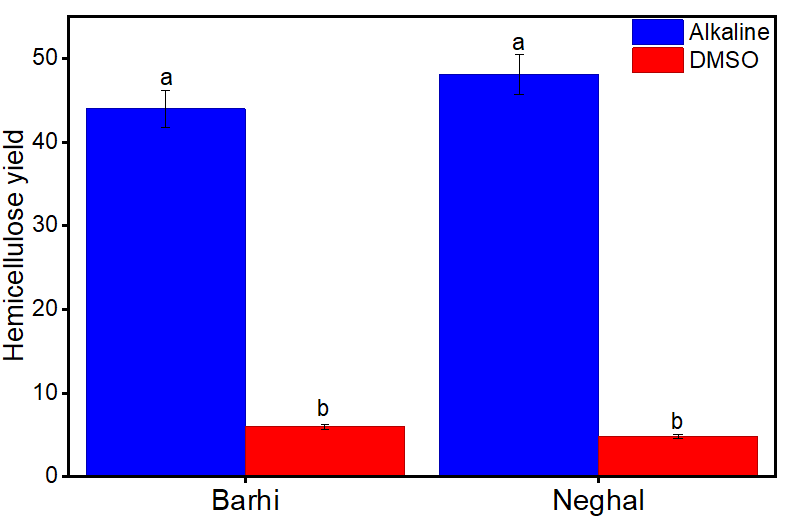
**

**Figure S1.** Hemicellulose yields from alkaline (20%, 8 h, 60^o^C) and DMSO extractions for Barhi and Neghal. Different letters between bars indicate significant differences (p<0.05)

**Figure S2.** ^1^H NMR of BH (Barhi hemicellulose extracted at 5%/6 h/60^o^C)

**Figure S3**. ^13^C NMR of BH (Barhi hemicellulose extracted at 5%/6 h/60^o^C)

**Figure S4.** ^1^H-^1^H NMR COSY of BH (Barhi hemicellulose extracted at 12.5%/8 h/25^o^C)

**Figure S5.** ^1^H-^1^H NMR TOCSY of BH (Barhi hemicellulose extracted at 5%/6 h/60^o^C)

**Figure S6.** ^1^H-^13^C NMR HSQC of BH (Barhi hemicellulose extracted at 5%/6 h/60^o^C)

**Figure S7.** ^1^H-^13^C NMR HMBC of BH (Barhi hemicellulose extracted at 5%/6 h/60^o^C)

**Figure S8.** ^1^H NMR of BR (Barhi hemicellulose extracted at 12.5%/8 h/25^o^C)

**Figure S9.** ^1^H-^1^H NMR COSY of BR (Barhi hemicellulose extracted at 12.5%/8 h/25^o^C)

**Figure S10.** ^1^H-^13^C NMR HSQC of BR (Barhi hemicellulose extracted at 12.5%/8 h/25^o^C)

**Figure S11.** ^1^H NMR of NH (Neghal hemicellulose extracted at 5%/6 h/60^o^C)

**Figure S12.** ^1^H-^13^C NMR HSQC of NH (Neghal hemicellulose extracted at 5%/6 h/60^o^C)

**Figure S13.** ^1^H NMR of NR (Neghal hemicellulose extracted at 12.5%/8 h/25^o^C)

**Figure S14.** ^1^H-^1^H NMR COSY of NR (Neghal hemicellulose extracted at 12.5%/8 h/25^o^C)

**Figure S15.** ^1^H-^1^H NMR TOCSY of NR (Neghal hemicellulose extracted at 12.5%/8 h/25^o^C)

**Figure S16.** ^1^H-^13^C NMR HSQC of NR (Neghal hemicellulose extracted at 12.5%/8 h/25^o^C)
